# Epithelioid hemangioma of the colon: a case report

**DOI:** 10.1590/S1516-31802008000500011

**Published:** 2008-09-04

**Authors:** Ronaldo Nonose, Denise Gonçalves Priolli, Izilda Aparecida Cardinalli, Felipe Rodrigues Máximo, Patrícia Savói Pires Galvão, Carlos Augusto Real Martinez

**Keywords:** Colon, Hemangioma, Obesity, Angiolymphoid hyperplasia with eosinophilia, Vascular neoplasm, Colorectal cancer, Cólon, Hiperplasia angiolinfóide com eosinofilia, Neoplasias vasculares, Câncer colorretal

## Abstract

**CONTEXT:**

Epithelioid hemangioma or angiolymphoid hyperplasia with eosinophilia is an uncommon benign vascular neoplasm that is usually located on the face or neck. Exceptionally, it has been described affecting the colon, with only two such cases described in the worldwide literature. The aim here was to present a case of primary epithelioid hemangioma of the sigmoid colon with confirmation by immunohistochemical examination.

**CASE REPORT:**

A 37-year-old woman had had a complaint of intermittent abdominal pain for six months. Two months after the condition started, she began to present changes in her intestinal habit, with evacuations containing blood and mucus and a weight loss of 4 kg over this period. At physical examination, a palpable mass was noted in the lower left quadrant of the abdomen. Neoplasia of the colon was clinically suspected and she underwent colonoscopy. This demonstrated the presence of a vegetating sessile lesion of approximately 5 cm in diameter, at a distance of 36 cm from the anal margin. It occupied 80% of the intestinal lumen. A biopsy collected during the examination suggested a diagnosis of neoplasia of vascular origin. After surgical resection, histopathological examination of the resected specimen confirmed the diagnosis of epithelioid hemangioma of the colon, which was backed up by the immunohistochemical panel (factor VIII, Ki-67, CD-34). At present, three years after the surgery, the patient is asymptomatic, she has recovered her normal weight and she has normal findings from control colonoscopy. Despite the rarity of neoplasia of vascular origin, this possibility should be considered in the differential diagnosis for colorectal tumors.

## RESUMO

**CONTEXTO:**

Hemangioma epitelióide ou hiperplasia angiolinfóide com eosinofilia são neoplasias vasculares benignas raras, habitualmente localizadas na face e pescoço. O acometimento do intestino grosso é excepcionalmente descrito, existindo apenas dois casos descritos na literatura mundial. O objetivo deste artigo é apresentar um caso de hemangioma epitelióide primário do sigmóide com diagnóstico histopatológico confirmado por meio de estudo imunoistoquímico.

**RELATO DE CASO:**

Mulher de 37 anos apresentou queixa de dor abdominal de forte intensidade, intermitente, localizada no hipogástrio. Dois meses após o início do quadro, notou alteração do hábito intestinal, evacuações com sangue, muco e perda ponderal de 4 quilos no período. Ao exame físico abdominal, identificou-se massa palpável no quadrante inferior esquerdo. Com suspeita clínica de neoplasia de cólon foi submetida a colonoscopia, que demonstrou presença de lesão vegetante de aproximadamente cinco centímetros de diâmetro, ocupando cerca de 80% da luz colônica. A biópsia mostrou a presença de neoplasia de origem vascular. Após a ressecção cirúrgica, o exame histopatológico do espécime extirpado estabeleceu o diagnóstico de hemangioma epitelióide do cólon, confirmado por meio de painel imunoistoquímico (fator VIII, Ki-67, CD-34). No momento, a paciente encontra-se bem, tendo recuperado o peso inicial três anos após a cirurgia e apresenta resultado de colonoscopia de controle normal. Não obstante a raridade, deve-se considerar a possibilidade das neoplasias de origem vascular no diagnóstico diferencial dos tumores colorretais.

## INTRODUCTION

Epithelioid hemangioma (EH), also known as angiolymphoid hyperplasia with eosinophilia, is a lesion of vascular origin that was described for the first time in 1969.^1^ EHs are most frequently found in the skin and subcutaneous cellular tissue of the head, particularly around the ears.^2^ Involvement of abdominal viscera is a rare event but, if it occurs, the liver, spleen or small intestine are preferentially affected.^2^ It is exceptional for the colon to be affected and, to the best of our knowledge, only two cases have been described in the literature.^3^

The purpose of the present report was to present a case of primary EH of the colon with a histopathological diagnosis that was confirmed by an immunohistochemical panel.

## CASE REPORT

A 37-year-old woman had had a complaint of abdominal pain located in the hypogastrium for six months, with dysentery and changes in her intestinal habit. She had noted a weight loss of 4 kg since the start of the symptoms. A mobile mass of approximately 6 cm in diameter was palpated in the abdomen. Rectal examination showed the presence of blood and mucus.

Hematological examination demonstrated hemoglobin of 8.2 g/dl and a leukocyte count of 10,000 per mm^3^, but with a normal eosinophil count. The tumor markers CEA and Ca19-9 presented values of 1.1 ng/ml and 5.71 U/ml respectively and the anti-HIV serological test was negative.

**Figure 1:**
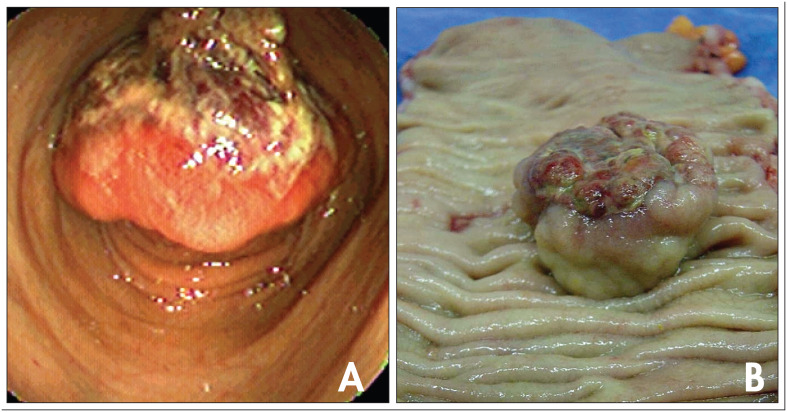
**A.**Endoscopic view of the sigmoid colon showing polypoid lesion occupying approximately 80% of the intestinal lumen. **B.** Longitudinal section along the sigmoid colon, showing polypoid tumor with central ulceration.

Colonoscopy demonstrated a vegetating polypoid lesion of 5 cm in diameter, 36 cm from the anal margin, which obstructed approximately 80% of the intestinal lumen (Figure 1A). The tumor presented a reddish surface coloration, with multiple small-sized ulcerations that alternated with areas of necrosis. Histopathological examination of the fragments obtained from biopsy showed a tumor of vascular origin, and the possibility that this might have been malignant could not be ruled out from the fragments examined. Because of the endoscopic characteristics of the lesion and the possibility of malignant neoplasia, it was decided not to perform endoscopic resection.

During laparotomy, a vegetating intraluminal lesion was found located in the sigmoid colon, which did not compromise the serosa layer and did not show regional lymphadenomegaly or metastases. It was decided to perform sigmoidectomy and lymphadenectomy. When the resected specimen from the colon was opened, it revealed a polypoid lesion of 5.5 cm in diameter, located 12 cm from the lower resection margin. It presented reddish coloration and fibroelastic consistency, with areas of ulceration and necrosis of the mucosa that were partially covered with fibrin, and with signs of recent hemorrhaging (Figure 1B). Sixteen lymph nodes from the excised segment were dissected.

**Figure 2:**
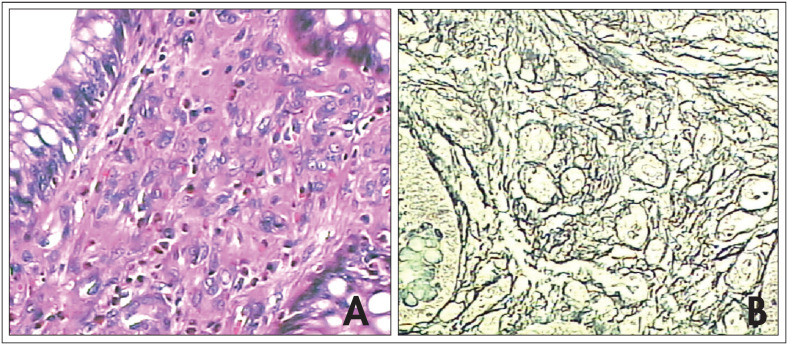
Epithelioid hemangioma of the sigmoid. **A.** Vascular proliferation separating the intestinal crypts (hematoxylin-eosin, 200 x). **B.** Staining by reticulin, showing the vascular nature of the lesion (reticulin, 200 x).

Analysis under a microscope using the hematoxylin-eosin technique showed neoplasia formed by intense proliferation of capillaries that were compactly grouped at depth and with weaker arrangement in the more superficial portions, thereby making the capillary lumen smaller inside the lesion. The intense vascular proliferation caused separation of the intestinal glands, although without invading them (Figure 2A). Endothelial proliferation on the vascular wall made the capillaries prominent, but without the presence of atypia. The endothelial cells had epithelioid characteristics, with acidophil cytoplasm, ovaloid or elongated nuclei and indistinct nucleoli. Amid this, the intense vascular proliferation drew attention to abundant infiltration of inflammatory cells consisting mainly of eosinophils and some lymphocytes. Special staining using reticulin was able to demonstrate that the characteristic architecture of the neoplasia was predominantly vascular (Figure 2B). The lymph nodes examined were found to be free from neoplastic involvement. An immunohistochemical panel for investigating the tissue expression of factor VIII and CD-34 presented intense expression, which showed the endothelial vascular nature of the neoplasm. Ki-67 cell proliferation factor levels demonstrated that there was little cell proliferative activity.

At present, three years after surgery, she no longer presents rectal bleeding and has recovered her initial weight. Imaging and endoscopic examinations carried out at the ends of the first and third years of follow-up did not show any signs of recurrence.

## DISCUSSION

EH with involvement of the colon is rare and represents only 0.001% of all colorectal tumors.^4^ In a review of the literature from 1960 onwards, only two cases of primary EH in the colon could be found.^3^ Like vascular neoplasia in other locations, vascular tumors in the colon are generally divided into three categories, based on their clinical evolution. The benign variant includes hemangiomas in all their histopathological presentations (epithelioid and cavernous).^3^ The intermediate variant is represented by hemangioendothelioma (epithelioid and hobnail).^2^ Angiosarcoma and Kaposi's sarcoma form the malignant variant.^2^

EH is considered to be a type of low-grade neoplasia, with the potential to progress. Local recurrence is found in one third of the cases, although with a low possibility of developing lymph node metastases or distant metastases.^2^ Although EH presents neoplastic characteristics, it has only been possible in 60% of the cases to demonstrate that damage to the wall of large vessels was present, and this has meant that the term EH is not completely accepted as a neoplastic entity.^2^

EH is often confounded with Kimura's disease because of superficial morphological similarities. However, in Kimura's disease, there is marked serum eosinophilia and hyperimmunoglobulinemia, at the cost of IgE and lymph node involvement.^4,5^ In the patient of the present report, it was not possible to find serum eosinophilia or hyperimmunoglobulinemia.

EH is most common between the second and fourth decades of life, with predominance among women.^2^ The lesion is generally polypoid and presents a reddish surface with areas of surface bleeding, particularly when subjected to trauma. Superficial ulceration with the formation of fibrin-leukocytic crusts is frequently identified. The hemorrhagic appearance of the external surface may often be interpreted as hemorrhagic necrosis of colorectal adenocarcinoma.

Histopathological examination shows abundant vascular proliferation with the formation of blood vessels of tortuous pattern.^2^ The endothelial cells have rounded or lobular nuclei and abundant acidophilic cytoplasm, containing occasional vacuoles that represent the formation of a primitive vascular lumen. Abundant infiltration of inflammatory cells consisting mainly of eosinophils and more rarely lymphocytes is frequently noted.^2^ In the patient of the present report, staining by means of the reticulin technique, specific for blood vessels, demonstrated a large quantity of fibers located between the neoplastic and eosinophilic cells. Immunohistochemical panels that include CD31, CD34 and the antigen associated with Factor VIII typically demonstrate positive immunostaining in tumors with endothelial differentiation.

The treatment for EH is eminently surgical, not only because of the diagnostic uncertainty, but also particularly because of the recurrent bleeding that often leads to anemia. More rarely, there may be large-scale hemorrhaging, which causes hemodynamic instability with the need for urgent surgical intervention.^3^ The prognosis for the disease, following surgical resection, is favorable, rarely followed by recurrence, as seen in the case of the patient of the present report.

## CONCLUSION

Despite the rarity of neoplasia of vascular origin, this possibility should be considered in the differential diagnosis for tumors of the colon.
